# A genomic approach to understand interactions between *Streptococcus pneumoniae* and its bacteriophages

**DOI:** 10.1186/s12864-015-2134-8

**Published:** 2015-11-18

**Authors:** Philippe Leprohon, Hélène Gingras, Siham Ouennane, Sylvain Moineau, Marc Ouellette

**Affiliations:** Centre de recherche en Infectiologie du Centre de Recherche du CHU de Québec, Université Laval, 2705 Boul. Laurier, Québec, QC Canada, G1V 4G2; Département de Microbiologie, Infectiologie et Immunologie, Faculté de Médecine, Université Laval, 1050, avenue de la Médecine, Québec, QC Canada, G1V 0A6; Département de Biochimie, Microbiologie et Bio-informatique and PROTEO, Faculté des Sciences et Génie, Université Laval, Québec, QC Canada; Félix d’Hérelle Reference Center for Bacterial Viruses and GREB, Faculté de Médecine Dentaire, Université Laval, Québec, QC Canada

**Keywords:** *Streptococcus pneumoniae*, Bacteriophage, Resistance, Whole genome sequencing, GntR regulator

## Abstract

**Background:**

Bacteriophage replication depends on bacterial proteins and inactivation of genes coding for such host factors should interfere with phage infection. To gain further insights into the interactions between *S. pneumoniae* and its pneumophages, we characterized *S. pneumoniae* mutants selected for resistance to the virulent phages SOCP or Dp-1.

**Results:**

*S. pneumoniae* R6-SOCP^R^ and R6-DP1^R^ were highly resistant to the phage used for their selection and no cross-resistance between the two phages was detected. Adsorption of SOCP to R6-SOCP^R^ was partly reduced whereas no difference in Dp-1 adsorption was noted on R6-DP1^R^. The replication of SOCP was completely inhibited in R6-SOCP^R^ while Dp-1 was severely impaired in R6-DP1^R^. Genome sequencing identified 8 and 2 genes mutated in R6-SOCP^R^ and R6-DP1^R^, respectively. Resistance reconstruction in phage-sensitive *S. pneumoniae* confirmed that mutations in a GntR-type regulator, in a glycerophosphoryl phosphodiesterase and in a Mur ligase were responsible for resistance to SOCP. The three mutations were additive to increase resistance to SOCP. In contrast, resistance to Dp-1 in R6-DP1^R^ resulted from mutations in a unique gene coding for a type IV restriction endonuclease.

**Conclusion:**

The characterization of mutations conferring resistance to pneumophages highlighted that diverse host genes are involved in the replication of phages from different families.

**Electronic supplementary material:**

The online version of this article (doi:10.1186/s12864-015-2134-8) contains supplementary material, which is available to authorized users.

## Background

*Streptococcus pneumoniae* is an opportunistic colonizer of the nasopharynx and the causative agent of many serious diseases such as pneumonia, sepsis, meningitis and otitis media. Initially, strains of *S. pneumoniae* were exquisitely susceptible to penicillin, and β-lactam antibiotics were the recommended empirical treatment against pneumococcal diseases. However, pneumococci resistant to β-lactams and other classes of antibiotics now represent a major burden due to the spread of multidrug resistant clones [1–3] and penicillin-resistant pneumococci are listed among the most serious antibiotic resistance threats [4]. Antimicrobial resistance will require innovation not only in the development of new antibiotics but also in alternative treatment strategies and, in this context, biological therapeutics were included among the seven key areas of antimicrobial resistance for which research is urgently needed [5].

Bacteriophage (phage) therapy represents one of the promising alternatives against multidrug resistant pathogens. Strategies include the use of isolated virions but also some of their products like endolysins, a family of peptidoglycan hydrolases released at the terminal stage of phages replication cycle for the lysis of infected cells and phage progeny release. In the case of *S. pneumoniae*, a number of studies have demonstrated the potential of phage-produced endolysins against otitis media [6], bacteremia [7] and pneumonia [8, 9]. In contrast, while pneumophages have been repeatedly described in the literature [10–18], studies evaluating the use of whole virions have lagged behind. A high proportion of *S. pneumoniae* clinical isolates are lysogens [19–21] and the vast majority of pneumophages currently identified are temperate phages. However, because temperate phages have the ability to transfer host DNA and/or increase virulence [22], virulent phages are thought to be better suited for biocontrol purposes.

Very few virulent bacteriophages infecting *S. pneumoniae* have been isolated. Despite the isolation of Omega phages several decades ago [18], the pneumophages Dp-1 and Cp-1 are the only lytic phages that remain available in curated bacteriophage collections. Phage Dp-1 was the first virulent pneumophage to be isolated [12]. It belongs to the *Siphoviridae* family and has a DNA genome of 56,506 bp coding for 72 putative proteins, 39 of which could be annotated based on sequence homology [23]. Phage Cp-1 was isolated in 1981 [16] and is a member of the *Podoviridae* family. Its DNA genome of 19,345 bp contains 25 open reading frames >50 nucleotides of which a third could be assigned a function based on sequence homology at the protein level with gene products from bacteriophage phi29 infecting *Bacillus subtilis* [24]. The infectivity of Dp-1 and Cp-1 was shown to require choline in the pneumococcus cell wall [12]. More recently, a natural variant of Cp-1 called SOCP with a genome of 19,347 bp and 31 single nucleotide variations has been described [25]. The annotation of the genome of SOCP revealed 27 open reading frames, each preceded by a putative ribosome-binding site, and a putative function could be assigned to 12 proteins [25].

One of the perceived drawbacks of phage therapy is the likely emergence of phage-resistant derivatives or clones [26]. Such phage resistance phenotype may be due to dedicated defense mechanisms harboured by some strains, including restriction-modification systems and CRISPR-Cas systems or may be due to the absence of specific host factors such as phage receptors or to the presence of interfering capsular polysaccharides [27]. In addition to phage adsorption at the cell surface, many other steps of the phages lytic cycle such as replication, transcription and translation also likely depend on bacterial cytoplasmic gene products. The absence or inactivation of some of these genes could prevent the lysis of phage-infected cells. Therefore, an in-depth understanding of the bacterial factors involved in phage-host interactions is needed to optimize the selection of appropriate therapeutic phage.

Here, we identified host factors involved in the pneumophage infection process. We first confirmed, using molecular tools, the role of the capsule in protecting pneumophage infection. We also infected the unencapsulated *S. pneumoniae* R6 with virulent phage Dp-1 and SOCP and selected spontaneous bacteriophage-insensitive mutants. Genome sequencing of the mutants and functional analysis revealed diverse mutations implicated in resistance to pneumophages.

## Results

### Interaction of bacteriophages SOCP and DP-1 with S. pneumoniae

**Fig. 1:**
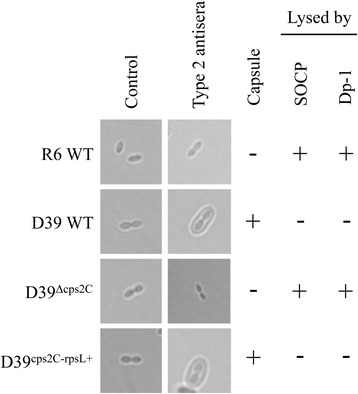
Detection of type 2 capsular polysaccharides in *S. pneumoniae*. Preparations of *S. pneumoniae* R6 WT and *S. pneumoniae* D39 WT, or from D39^Δcps2C^ and its complemented D39^cps2C-rpsL^ version with no antiserum (control) and with type 2 antisera were viewed under oil immersion using a Nikon Eclipse TE300 microscope and a 100× objective. A positive Quellung reaction is observed when the cells appear swollen in the presence of antisera, which is indicative of the presence of type 2 capsular polysaccharides. The presence and absence of the capsule is indicated by a ‘+’ and a ‘-’, respectively. Sensitivity and resistance to pneumophages (SOCP and Dp-1) is indicated by a ‘+’ and a ‘-’, respectively

The unencapsulated strain *S. pneumoniae* R6 is highly sensitive to pneumophages SOCP and Dp-1 (Table 1). This is in sharp contrast to its encapsulated *S. pneumoniae* D39 ancestor which demonstrates complete resistance (Table 1). The pneumococcal capsule had previously been shown to inhibit infection by omega pneumophages [27] and it is possible that a similar protective role also occur against bacteriophages SOCP and Dp-1. The role of the pneumococcal capsule in the resistance to phages SOCP and Dp-1 was assessed by generating a *S. pneumoniae* D39 derivative inactivated for the gene *cps2C* (SPD_0317). The inactivation of *cps2C* was previously shown to result in a severe reduction in capsule size [28]. Capsular serotyping with an antisera directed against type 2 pneumococcal capsular polysaccharides confirmed the absence of detectable capsule in *S. pneumoniae* D39^Δcps2C^ (Fig. 1). *S. pneumoniae* D39^Δcps2C^ also had the same level of sensitivity to SOCP and Dp-1 as *S. pneumoniae* R6 (Table 1). Reverting *cps2C* to a WT version in D39^Δcps2C^ restored the capsule and abrogated phage sensitivity in the resulting *S. pneumoniae* D39^cps2C-rpsL^ transformant (Fig. 1 and Table 1). The pneumococcal serotype 2 capsule is thus a major determinant of resistance against virulent pneumophages.

**Table 1:** Sensitivity of *S. pneumoniae* strains to bacteriophages SOCP and Dp-1

*S. pneumoniae* strains	*cps2C* status^a^	Capsule^b^	Phages			
			SOCP		Dp-1	
			Phage titer (PFU/mL)	EOP^c^	Phage titer (PFU/mL)	EOP^c^
R6	*NA*	-	10^9^	1	10^10^	1
D39	WT	+	0	*NA*	0	*NA*
D39^Δcps2C^	KO	-	10^9^	1	10^10^	1
D39^cps2C-rspL^	WT	+	0	*NA*	0	*NA*
R6-SOCP^R^	*NA*	-	1	10^−9^	10^10^	1
R6-DP1^R^	*NA*	-	10^9^	1	10^2^	10^−8^

^a^WT, *S. pneumoniae* D39 wild type allele; KO, *S. pneumoniae* D39 allele inactivated by insertion-duplication mutagenesis; *NA*, Not applicable since the gene is absent in *S. pneumoniae* R6 due to the deletion of the capsule locus [77, 78]

^b^‘+’ and ‘-’ respectively indicate the presence and absence of the pneumococcal capsule as determined by the Quellung reaction (see Fig. 1)

^c^EOP, efficiency of plaquing. Represents the ratio of phage titers from the test strain to the indicator strain *S. pneumoniae* R6 WT. Measured from three independent triplicates. *NA*, not applicable because of absolute bacteriophage resistance

To further our understanding of interactions between pneumophages and *S. pneumoniae* R6, we selected bacteriophage-insensitive mutants (see Methods). One mutant resistant to phage SOCP and another resistant to phage Dp-1 were chosen for further characterization and these were named *S. pneumoniae* R6-SOCP^R^ and R6-DP1^R^, respectively (Table 1). The bacteriophage-insensitive mutants R6-SOCP^R^ and R6-DP1^R^ mutants had unaltered growth kinetics compared to *S. pneumoniae* R6 wild-type (WT) (Additional file 1). Both mutants also displayed a normal morphology under electronic microscopy and had unaltered cell wall thickness compared to their *S. pneumoniae* R6 parent (Additional file 2). The R6-SOCP^R^ and R6-DP1^R^ mutants were highly resistant to the phage used for their selection. There were no detectable plaques when a SOCP bacteriophage suspension (10^9^ PFU/mL) was spotted onto a lawn of R6-SOCP^R^ (Table 1). In contrast, spotted SOCP bacteriophages onto a lawn of *S. pneumoniae* R6 WT resulted in a confluent zone of clearing (Table 1). Similarly, the efficiency of plaquing (EOP) of Dp-1 on R6-DP1^R^ was determined to 10^−8^ when compared to the indicator strain *S. pneumoniae* R6 (Table 1). The mutants R6-SOCP^R^ and R6-DP1^R^ remained sensitive to Dp-1 and SOCP, respectively (Table 1).

### Adsorption and replication of pneumophages SOCP and Dp-1

**Fig. 2:**
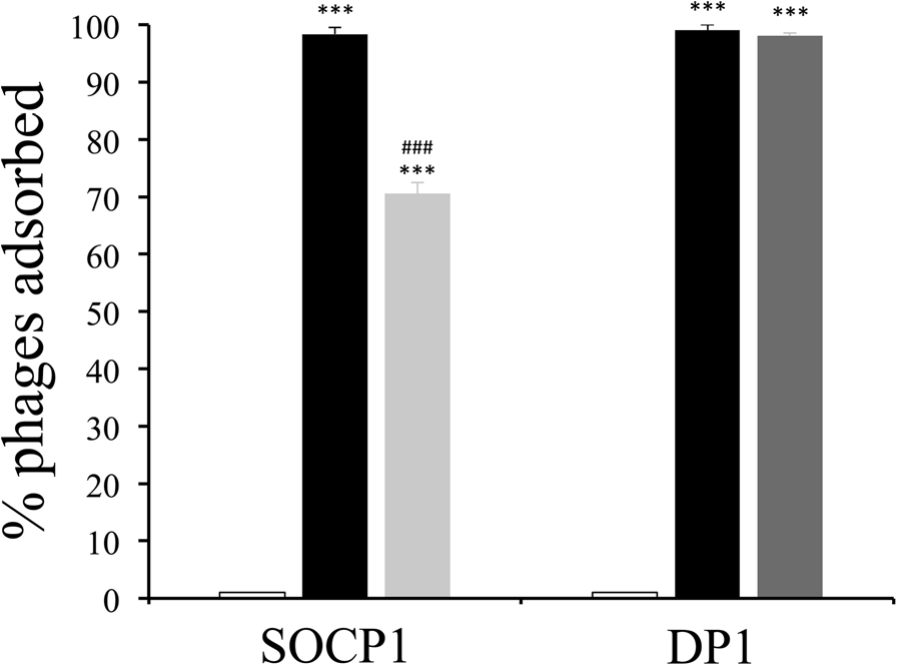
Adsorption of phages SOCP and Dp-1 on *S. pneumoniae* strains. Rates of adsorption of SOCP and Dp-1 on *S. pneumoniae* R6 WT (black), R6-SOCP^R^ (light grey) or R6-DP1^R^ (dark grey). No adsorption occurred in the absence of bacteria (empty bar). Adsorption of SOCP on R6-DP1^R^ and adsorption of Dp-1 on R6-SOCP^R^ was not measured since no bacteriophage cross-resistance occurred in the mutants (see Table 1). ^***^denotes significant differences in adsorption compared to the mock control (*p* < 0.0001; one-way non-parametric ANOVA). ^###^denotes significant differences in adsorption compared to R6 WT (*p* < 0.0001; one-way non-parametric ANOVA)

To gain further insights into the step of the infective cycle that is blocked in the resistant mutants, we first tested whether bacteriophage adsorption was prevented in R6-SOCP^R^ and R6-DP1^R^. Adsorption assays showed that pneumophages SOCP and Dp-1 efficiently adsorbed to *S. pneumoniae* R6 but that adsorption of SOCP was reduced on mutant R6-SOCP^R^ (Fig. 2). SOCP adsorption levels on R6-SOCP^R^ still remain substantially high however (Fig. 2), suggesting additional mechanisms for resistance (Table 1). In contrast, the *S. pneumoniae* R6-DP1^R^ mutant adsorbed DP-1 bacteriophages as efficiently as *S. pneumoniae* R6 (Fig. 2). The resistance of both mutants thus likely affect a step of the lytic cycle beyond the phage adsorption process.

**Fig. 3:**
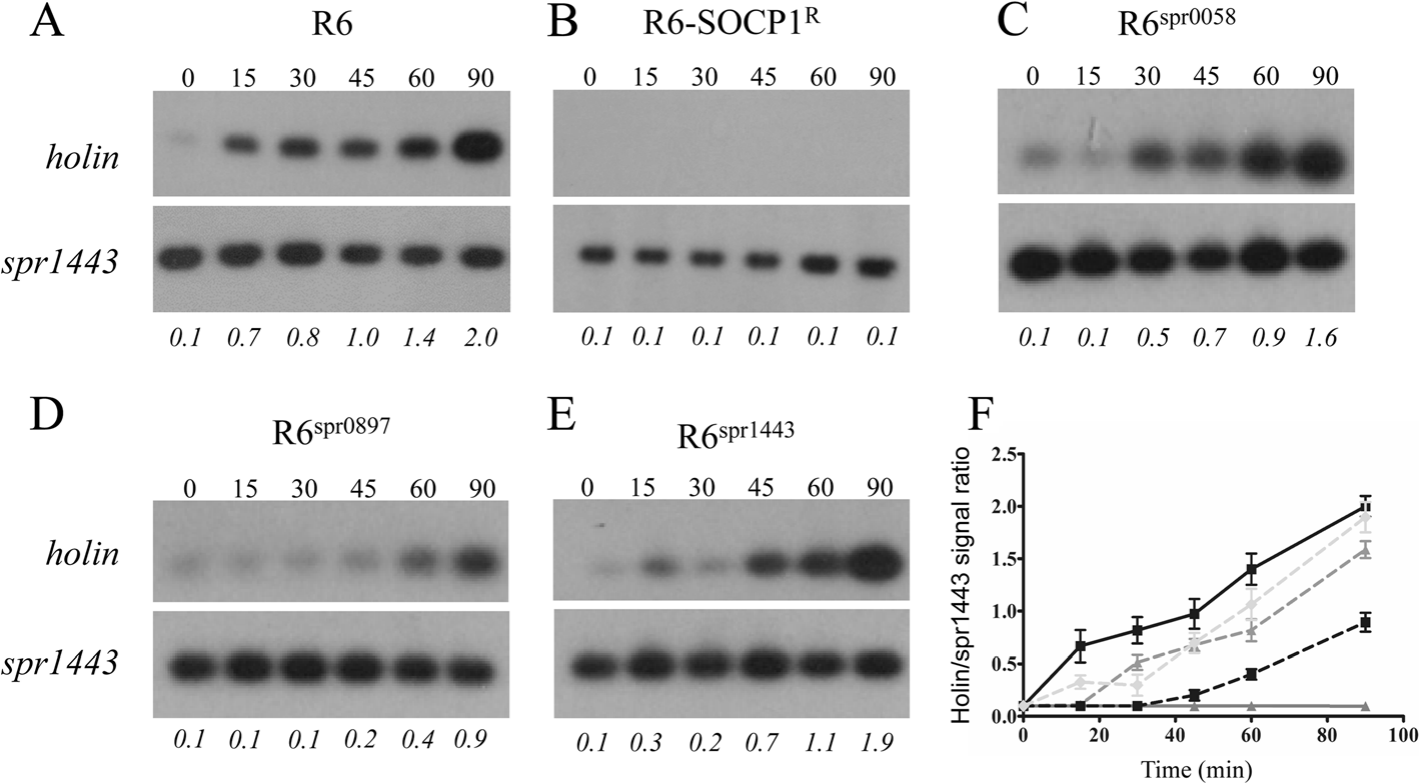
Kinetics of SOCP DNA replication. *S. pneumoniae* R6 WT (**a**), R6-SOCP^R^ (**b**) and *S. pneumoniae* R6 transformed with R6-SOCP^R^-derived alleles for genes spr0058 (**c**), spr0897 (**d**) or spr1443 (**e**) were infected with the lytic phage SOCP at a MOI of 0.1. Total DNA was extracted at baseline and at 15, 30, 45, 60 and 90 min after infection. Total DNA was digested with SspI before being hybridized with an [α-^32^P]dCTP-labeled probe covering the holin gene of SOCP (top blots). DNA loading was controlled by hybridizing the blots with an [α-^32^P]dCTP-labeled probe covering the spr1443 gene from *S. pneumoniae* R6 (bottom blots). Holin/spr1443 signal ratios are indicated in italics below the blots. Hybridizations were done in triplicates and representative blots are shown. **f** Plot of holin/spr1443 signal ratios for *S. pneumoniae* R6 WT (black), R6-SOCP^R^ (dark grey), R6^spr0058^ (dashed dark grey), R6^spr0897^ (dashed black), and R6^spr1443^ (dashed light grey). For each strain, the ratios at each time point (except for R6^spr1443^ at 90 min) were significantly different (*p* < 0.01 by two-way ANOVA) than those of R6 WT

**Fig. 4:**
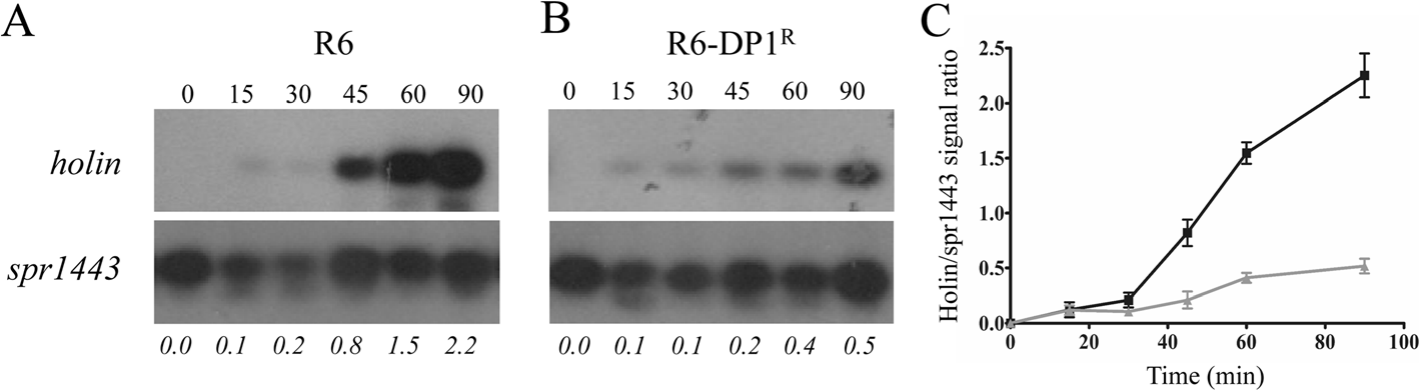
Kinetics of Dp-1 DNA replication. *S. pneumoniae* R6 WT (**a**) and R6-DP1^R^ (**b**) were infected with the lytic phage Dp-1 at a MOI of 0.1. Total DNA was extracted at baseline and at 15, 30, 45, 60 and 90 min after infection. Total DNA was digested with SspI before being hybridized with an [α-^32^P]dCTP-labeled probe covering the holin gene of DP-1. DNA loading was controlled by hybridizing the blots with an [α-^32^P]dCTP-labeled probe covering the spr1443 gene from *S. pneumoniae* R6. Holin/spr1443 signal ratios are indicated in italics below the blots. Hybridizations were done in triplicates and representative blots are shown. **c** Plot of holin/spr1443 signal ratios for *S. pneumoniae* R6 WT (black) and R6-DP1^R^ (dark grey). The R6-DP1^R^ ratios from 45 to 90 min were significantly different (*p* < 0.001 by two-way ANOVA) than those of R6 WT

We next assayed whether the intracellular replication of pneumophages is impaired in *S. pneumoniae* R6-SOCP^R^ and R6-DP1^R^. Total DNA (including chromosomal DNA and phage DNA) was extracted at successive time points following infection of *S. pneumoniae* R6-SOCP^R^ and R6-DP1^R^ with phages SOCP and Dp-1, respectively. The DNA samples were then digested with SspI before being electrophoresed and transferred on Nylon membrane. Hybridizing membranes with probes covering the holin gene from SOCP or Dp-1 allowed monitoring the kinetics of phage genome replication by measuring the intensity of phage DNA over time after infection. In *S. pneumoniae* R6, replication of SOCP was already well advanced at 15 min following infection and SOCP DNA levels had increased by an estimated 20-fold after 90 min (Fig. 3a and f). In contrast, no holin-derived signals could be detected following infection of R6-SOCP^R^ even at the 90 min time point (Fig. 3b and f). This suggests that resistance in *S. pneumoniae* R6-SOCP^R^ occurs at very early stages of the infection cycle. In our experimental conditions, the replication of pneumophage Dp-1 within *S. pneumoniae* R6 began after a latency of about 30 to 45 min [29], and after 90 min Dp-1 DNA levels had increased by an estimated 22-fold (Fig. 4a and c). An increase in Dp-1 holin signals was also observed upon infection of *S. pneumoniae* R6-DP1^R^ but at a 4-fold decreased rate compared to *S. pneumoniae* R6 (Fig. 4b-c). This suggests that Dp-1 replication occurred but was severely impaired in the R6-DP1^R^ mutant.

### Whole genome sequencing of S. pneumoniae R6-SOCP^R^ and R6-DP1^R^

Whole genome sequencing is useful for identifying genes involved in host-phage interactions [30–32] and the genomes of *S. pneumoniae* R6-SOCP^R^ and R6-DP1^R^ (one clone of each) were sequenced to identify mutations putatively implicated in resistance. Whole-genome sequencing revealed a total of eleven and five nucleotide mutations in the genomes of R6-SOCP^R^ and R6-DP1^R^, respectively (Table 2). None of the mutations (or mutated genes) was common to both mutants (Table 2) which is consistent with the absence of cross-resistance (Table 1). Every mutation detected in R6-SOCP^R^ occurred within coding sequences (in eight genes) while two of those found in R6-DP1^R^ were non-coding (Table 2). For both mutants, mutations in open reading frames invariably led to amino acid substitutions (Table 2).

**Table 2:** List of all mutations identified in the genome of R6-SOCP^R^ and R6-DP1^R^

Strains	Genes	Function	Mutations^a,b,c^	
R6-SOCP^R^	spr0058	GntR-type transcription factor	**G460A**	
			***E154K***	
	spr0093	Phosphoglycolate phosphatase	G343T	
			*D115Y*	
	spr0897	Glycerophosphoryl diester phosphodiesterase	**C484A**	
			***P162T***	
	spr1191	ABC protein – ATP binding domain	G1624T^d^	
			T1625G^d^	G1629T
			*V542C*	*L543F*
	srp1443	Mur ligase homolog	**T1223C**	
			***V408A***	
	spr1584	Hypothetical protein	A1229C	
			*Y410S*	
	spr1777	RNA polymerase rpoB	G827C	
			*R276P*	
	spr1923	Hypothetical protein	G690C	G691C
			*Q230H*	*A231P*
R6-DP1^R^	spr0291	Phosphotransferase system sugar-specific EII component	C-101G	
	spr1130	McrB subunit of McrBC restriction endonuclease	**T985G** ^d^	
			**G986C** ^d^	
			***C329A***	
	spr1445	Dipeptidase M24 family	G918A	
			*M306I*	
	spr1453	Major facilitator transporter	T-187G	

^a^For each mutation, the change at the nucleotide level is indicated on top and the corresponding substitution at the amino acid level is indicated in italic underneath

^b^Mutations in bold have a confirmed role in resistance

^c^For mutations in intergenic regions, an hyphen in front of the mutated position indicates the nucleotide position upstream of the ATG

^d^Both mutated positions are part of the same codon and lead to a single amino acid substitution

The role of each mutation in pneumophage resistance was assessed by resistance construction, a strategy shown useful for pinpointing mutations implicated in antibiotic resistance in *S. pneumoniae* [33–39]. This was done by transforming *S. pneumoniae* R6 WT with separate PCR products covering variant alleles derived from R6-SOCP^R^ or R6-DP1^R^ along with a PCR fragment covering the *rpsL* allele of *S. pneumoniae* CP1250 [40] and conferring resistance to streptomycin. The latter was used as a surrogate marker for the selection of transformants. The *rpsL* allele had no impact on pneumophage susceptibility levels when transformed alone (Tables 3 and 4). The streptomycin-resistant transformants were then screened for the presence of mutated alleles. Using a similar approach, the mutated genes were reverted back to their WT versions in the R6-SOCP^R^ or R6-DP1^R^ mutants for phenotype confirmation. Finally, each reconstructed strain was tested for resistance to SOCP or Dp-1.

**Table 3:** The role of mutations detected in R6-SOCP^R^ in resistance to phage SOCP

Strains^a^	Alleles^b^								EOP^c^
	spr0058	spr0093	spr0897	spr1191	srp1443	spr1584	spr1777	spr1923	(SOCP)
R6	WT	WT	WT	WT	WT	WT	WT	WT	1
R6^smR^	WT	WT	WT	WT	WT	WT	WT	WT	1
R6-SOCP^R^	R	R	R	R	R	R	R	R	10^−9^
R6^spr0058^	R	WT	WT	WT	WT	WT	WT	WT	10^−3^
R6^Δspr0058^	none^d^	WT	WT	WT	WT	WT	WT	WT	10^−3^
R6^spr0093^	WT	R	WT	WT	WT	WT	WT	WT	1
R6^spr0897^	WT	WT	R	WT	WT	WT	WT	WT	10^−3^
R6^spr1191^	WT	WT	WT	R	WT	WT	WT	WT	1
R6^spr1443^	WT	WT	WT	WT	R	WT	WT	WT	10^−3^
R6^spr1584^	WT	WT	WT	WT	WT	R	WT	WT	1
R6^spr1777^	WT	WT	WT	WT	WT	WT	R	WT	1
R6^spr1923^	WT	WT	WT	WT	WT	WT	WT	R	1
R6^spr0058-0897^	R	WT	R	WT	WT	WT	WT	WT	10^−9^
R6^spr0058-1443^	R	WT	WT	WT	R	WT	WT	WT	10^−9^
R6^spr0897-1443^	WT	WT	R	WT	R	WT	WT	WT	10^−9^
R6^spr0058-0897-1443^	R	WT	R	WT	R	WT	WT	WT	10^−9^
R6-SOCP^R_spr0058WT^	WT	R	R	R	R	R	R	R	10^−6^
R6-SOCP^R_spr0093WT^	R	WT	R	R	R	R	R	R	1
R6-SOCP^R_spr0897WT^	R	R	WT	R	R	R	R	R	10^−6^
R6-SOCP^R_spr1191WT^	R	R	R	WT	R	R	R	R	1
R6-SOCP^R_spr1443WT^	R	R	R	R	WT	R	R	R	10^−6^
R6-SOCP^R_spr1584WT^	R	R	R	R	R	WT	R	R	1
R6-SOCP^R_spr1777WT^	R	R	R	R	R	R	WT	R	1
R6-SOCP^R_spr1923WT^	R	R	R	R	R	R	R	WT	1

^a^R6^smR^ integrated an *rpsL* allele conferring resistance to streptomycin (smR). Each mutated and WT alleles presented in the table was co-transformed with this *rpsL* allele that was used as a surrogate marker for the selection of transformants (see Methods). Although not indicated, every transformants in Table 3 are smR

^b^WT, *S. pneumoniae* R6 WT gene sequence; R, *S. pneumoniae* R6-SOCP^R^ gene sequence

^c^EOP, efficiency of plaquing. Represents the ratio of phage titers from the test strain to the indicator strain R6 WT. Measured from three independent triplicates

^d^The gene spr0058 has been inactivated by insertion-duplication mutagenesis in this strain

**Table 4:** The role of mutations detected in R6-DP1^R^ in resistance to phage Dp-1

Strains^a^	Alleles^b^				EOP^c^
	spr0290	spr1130	spr1445	spr1453	(Dp-1)
R6	WT	WT	WT	WT	1
R6^smR^	WT	WT	WT	WT	1
R6-DP1^R^	R	R	R	R	10^−8^
R6^spr1130^	WT	R	WT	WT	10^−8^
R6^spr1445^	WT	WT	R	WT	1
R6-DP1^R^-^spr1130WT^	R	WT	R	R	1

^a^R6^smR^ integrated an *rpsL* allele conferring resistance to streptomycin (smR). Each mutated and WT alleles presented in the table was co-transformed with this *rpsL* allele that was used as a surrogate marker for the selection of transformants (see Methods). Although not indicated, every transformants in Table 4 are smR

^b^WT, *S. pneumoniae* R6 WT gene sequence; R, *S. pneumoniae* R6-DP1^R^ gene sequence

^c^EOP, efficiency of plaquing. Represents the ratio of phage titers from the test strain to the indicator strain R6 WT. Measured from three independent triplicate

### Mutations involved in resistance to SOCP

A role in resistance to phage SOCP was confirmed for three of the eleven mutations (in genes spr0058, spr0897 and spr1443) detected in the *S. pneumoniae* R6-SOCP^R^ mutant (Table 3). The gene spr0058 codes for a GntR-type transcription factor and the introduction of the spr0058 allele from R6-SOCP^R^ into *S. pneumoniae* R6 WT decreased the EOP of SOCP by three orders of magnitude (R6^spr0058^ in Table 3). Transforming *S. pneumoniae* R6 WT with PCR fragments covering the genes spr0897 (coding for a glycerophosphoryl phosphodiesterase) or spr1443 (coding for a Mur ligase homolog) amplified from *S. pneumoniae* R6-SOCP^R^ similarly decreased the EOP of SOCP by three logs (R6^spr0897^ and R6^spr1443^ in Table 3). In all cases, reverting any of the three mutations in *S. pneumoniae* R6-SOCP^R^ to a WT allele conferred coherent sensitivity levels to phage SOCP for the transformants R6-SOCP^R_spr0058WT^, R6-SOCP^R_spr0897WT^ and R6-SOCP^R_spr1443WT^ (Table 3). Moreover, introducing the spr0058, spr0897 and spr1443 mutations altogether in *S. pneumoniae* R6 produced transformants as resistant to SOCP as the original R6-SOCP^R^ mutant (R6^spr0058-0897-1443^ in Table 3), although the combination of any two mutations appears sufficient to confer high-level resistance (Table 3). Interestingly, introducing the spr0058, spr0897 or spr1443 mutations in *S. pneumoniae* R6 WT also impaired the DNA replication of SOCP (Fig. 3c-f), especially in the case of spr0897 (Fig. 3f).

When additional *S. pneumoniae* R6 mutants made resistant to SOCP were tested for the presence of mutations in spr0058, spr1897 and spr1443, three (out of four) additional mutants had a mutation in at least one of the genes (Table 5). None of the mutants had the exact same genotype for the three genes tested which preclude multiple sampling of the same end-point clone from the original culture (although divergence from a common ancestor mutated for spr1443 cannot be excluded) (Table 5). Instead, it highlights the major role of genes on the infective cycle of SOCP. For spr0897 and spr1443 the mutations even targeted the same amino acid as in the R6-SOCP^R^ mutant, leading to a different substitution in the case of spr0897 (Table 5). *S. pneumoniae* R6-SOCP-5^R^ was the only additional mutant with a mutation in gene spr0058, harbouring a non-sense mutation at codon 131 (Table 5). The E154K mutation originally detected in spr0058 in R6-SOCP^R^ (Table 2) is also expected to considerably alter the activity of the protein given that *S. pneumoniae* R6^spr0058^ harbouring the spr0058 E154K mutation from R6-SOCP^R^ displayed the same level of resistance to SOCP than *S. pneumoniae* R6^Δspr0058^ in which we inactivated spr0058 by insertion-duplication mutagenesis (Table 3). The gene product of spr0058 has similarity with regulators of the metabolite-responsive GntR family, which often regulate the expression of genes nearby of their location on the chromosome [41]. Comparative gene expression profiling by RNA-seq between *S. pneumoniae* R6 WT and *S. pneumoniae* R6^Δspr0058^ (Additional file 3) indeed revealed that an adjacent operon on the chromosome (spr0059-spr0065) coding for sugar transporters and metabolizing enzymes is overexpressed upon inactivation of spr0058 (Additional file 4). Additional putative operons also had their expression altered in *S. pneumoniae* R6^Δspr0058^, including several carbohydrate transport systems, which are also likely part of the spr0058 regulon (Additional file 4).

**Table 5:** Targeted screening for mutations in additional bacteriophage insensitive mutants

Strains^a^	Alleles^b,c^			
	spr0058	spr0897	spr1130	spr1443
R6-SOCP^R^	G460A	C484A	NA	T1223C
	*E154K*	*P162T*		*V408A*
R6-SOCP-2^R^	no mutation	no mutation	NA	no mutation
R6-SOCP-3^R^	no mutation	C485T	NA	T1223C
		*P162L*		*V408A*
R6-SOCP-4^R^	C393A	no mutation	NA	T1223C
	*stop*			*V408A*
R6-SOCP-5^R^	no mutation	no mutation	NA	T1223C
				*V408A*
R6-DP1^R^	NA	NA	T985G^d^	NA
			G986C^d^	
			*C329A*	
R6-DP1-2^R^	NA	NA	T985G^d^	NA
			G986C^d^	
			*C329A*	
R6-DP1-3^R^	NA	NA	T985G^d^	NA
			G986C^d^	
			*C329A*	
R6-DP1-4^R^	NA	NA	no mutation	NA
R6-DP1-5^R^	NA	NA	T985G^d^	NA
			G986C^d^	
			*C329A*	

^a^Five different mutants had initially been obtained from the same *S. pneumoniae* R6 parental culture for the selection of mutants insensitive to the phages SOCP or Dp-1

^b^For each mutation, the change at the nucleotide level is indicated on top and the corresponding substitution at the amino acid level is indicated in italic underneath

^c^NA, not applicable. These alleles were not sequenced because they should be irrelevant to the resistance phenotype of the phage

^d^Both mutated positions are part of the same codon and lead to a single amino acid substitution

### Mutations involved in resistance to Dp-1

Whole genome sequencing of *S. pneumoniae* R6-DP1^R^ revealed a total of five mutations, three of which occurred within open reading frames (Table 2). Resistance reconstruction further confirmed two nucleotide mutations targeting the same codon and leading to a single amino acid substitution in the McrB subunit of the McrBC restriction endonuclease (spr1130) (Table 2) were solely responsible for the high-level resistance of R6-DP1^R^ (Table 4). Indeed, pneumophage Dp-1 had the same EOP on R6-DP1^R^ than on *S. pneumoniae* R6^spr1130^, a *S. pneumoniae* R6 WT derivative into which a spr1130 PCR fragment amplified from R6-DP1^R^ was introduced (Table 4). Conversely, reverting spr1130 to a WT version in R6-DP1^R^ completely abrogated its resistance against pneumophage Dp-1 (R6-DP1^R_spr1130WT^ in Table 4), confirming the role of the mutation in the phage resistance phenotype. Testing for additional *S. pneumoniae* R6 mutants made resistant to Dp-1 again revealed that three out of the four additional mutants had the same mutation as R6-DP1^R^ (Table 5), although this time we cannot exclude that the same clone had been selected multiple times from the original culture.

## Discussion and conclusion

Bacteria have evolved diverse antiviral strategies to survive in phage-containing environments. These include adsorption resistance, which results in reduced interactions between the phage and its bacterium host; restriction-modification mechanisms and CRISPR-Cas systems, where bacteria survive and phage genomes are cleaved; and abortive infections, where bacteria die and phages usually remain trapped inside (reviewed in [42]). Also, many steps of the phage replication cycle likely depend on bacterial gene products, which if mutated may lead to phage resistance. Additional phage defence systems include superinfection exclusion whereby immunity occurs through the expression of a protein blocking the entry of DNA for specific phages. The genes encoding these proteins are often found in prophages, suggesting that in many cases these systems are important for phage–phage interactions rather than phage–host interactions (reviewed in [42]).

The genomic characterization of a *S. pneumoniae* R6 mutant insensitive to phage SOCP revealed mutations in genes spr0058, spr0897 and spr1443 that seemingly work additively to increase resistance. The gene spr0058 is coding for a transcriptional regulator of the GntR family and suffered from a G460A mutation leading to an E154K substitution in *S. pneumoniae* R6-SOCP^R^ (Table 2). GntR regulators are one of the most abundant and widely distributed groups of helix-turn-helix transcription factors [41]. They contain a DNA-binding domain at their N-terminus as well as an effector-binding and oligomerisation domain at the C-terminus of the protein in which the E154K mutation is located. The effector-binding domain is believed to modulate activity of bacterial transcription factors in response to binding small molecules [43]. The inactivation of spr0058 in R6^Δspr0058^ conferred the same SOCP resistance phenotype as the E154K mutation alone (Table 3) and it is tempting to speculate that the activity of the GntR regulator is also greatly impaired in the R6-SOCP^R^ mutant. Inactivation of GntR regulators through the acquisition of mutations was similarly shown to occur during adaption of *Comamonas testosteroni* to utilize phenol as the major carbon source, whereby several different missense mutations inactivated the repressor activity of the GntR regulator AphS [44]. GntR regulators bind DNA as dimers through interaction between their C-terminal domain [45] and one possibility is that the spr0058 mutation in R6-SOCP^R^ prevents repression by impairing with dimerization of the regulator at the operator-binding site. Another possibility would be that the mutation locks the repressor in a conformation mimicking the presence of bond ligand, thereby alleviating repression.

GntR family regulators often regulate (e.g. repress) the expression of neighbor genes [41] and the increased expression in R6^Δspr0058^ of adjacent genes spr0059-65 coding for sugar transporters and metabolizing enzymes is consistent with this assumption. Bacterial operons coding for carbohydrate transporters constitute functional units and, in addition to the transporter, they are usually coding for glycosyl-hydrolases for the production of mono- or disaccharides and/or enzymes for the metabolic steps linking specific sugars to glycolysis [46]. In the case of the spr0059-65 operon overexpressed in R6^Δspr0058^ (Additional file 4), the beta-galactosidase encoded by spr0059 was characterized as a surface enzyme responsible for cleavage of Galβ1-3GlcNac [47, 48] and it was proposed that the operon may thus code for a galactose uptake system [46]. Increased content of galactose in cell wall polymers have been correlated with increased bacteriophage resistance in *Lactococcus lactis* subsp. *cremoris* [49] and *Rhizobium meliloti* [50, 51]. In the case of phage-resistant *L. lactis subsp. cremoris*, an increase in galactosyl-containing lipoteichoic acid in the cell wall was further linked to a reduced bacteriophage adsorption [52]. Teichoic acid is also involved in pneumophage adsorption [53] and a similar phenomenon could possibly explain the reduced adsorption of SOCP on *S. pneumoniae* R6-SOCP^R^. The expression of many other genes (and operons) was altered in R6^Δspr0058^ besides spr0059-65 however (Additional file 4), and pinpointing the gene(s) actually implicated in resistance to SOCP will required further investigation.

The gene spr0897 mutated in R6-SOCP^R^ (Table 2) codes for a plasma membrane glycerophosphoryl diester phosphodiesterase (EC 3.1.4.46), an enzyme of the glycerophospholipid metabolism pathway involved in the production of glycerol-3-phosphate along with choline or ethanolamine from glycerophosphocholine or glycerophosphoethanolamine, respectively [54]. The P162T substitution in R6-SOCP^R^ is located at a conserved position within the second extracellular loop based on TMHMM transmembrane domains prediction. Most studies on glycerophosphoryl diester phosphodiesterases have focused on the catalytic domain which is located away from the P162T mutation at the C-terminus of the protein and the role of the mutation on the activity of the protein remains to be further explored. Given the role of these enzymes in the production of choline [55] and the dependency of pneumophage adsorption on choline-containing teichoic acid in the bacterial cell wall [53], it is possible that the mutation either interferes with the function of the protein or that it is favoring glycerophosphoethanolamine over glycerophosphocholine as its preferred substrate. In both cases this would translate into a decreased choline content (and also most likely of choline-binding proteins) in the cell wall, possibly explaining the decreased adsorption of SOCP on R6-SOCP^R^ (Fig. 2). However, glycerophosphoryl diester phosphodiesterases have been shown to influence gene expression [56] and an indirect role for the mutation in resistance by altering gene expression in R6-SOCP^R^ cannot be ruled out.

The last mutation implicated in resistance against bacteriophage SOCP in R6-SOCP^R^ occurred in gene spr1443 (Table 2) coding for a Mur ligase homolog named MurT [57–59]. MurT, along with the product of gene spr1444 (GatD), was recently shown to be responsible for the amidation of the glutamate residue in position 2 of the stem peptide of lipid II, a peptidoglycan precursor [57–59]. Amidation of lipid II is required for efficient peptidoglycan cross-linking in some Gram positive bacteria, including *S. pneumoniae*, and non-amidated glutamate-containing peptides were indeed found to be scarce in *S. pneumoniae* [60]. Cell wall cross-linking is important for optimal growth and influences susceptibility to antibiotics and murein hydrolases [57, 58]. The MurT V408A substitution detected in R6-SOCP^R^ is located at the C-terminus of the protein in a domain named DUF1727. This domain of unknown function is associated with the C-terminus of bacterial Mur ligases (http://pfam.xfam.org/family/PF08353). The role of the V408A substitution on the activity of MurT/GatD remains to be established but is unlikely to inactivate the activity of this amido transferase system which was shown to be essential in *S. aureus* [57, 58] and *S. pneumoniae* R6 [59].

Resistance to bacteriophage Dp-1 involved a single mutation in the gene spr1130 coding for the McrB subunit of a type IV McrBC restriction endonuclease (Tables 2 and 5). Type IV restriction endonucleases recognize modified, typically methylated, DNA. The McrBC endonucleases recognize and cleave DNA containing two hemi or fully methylated R^m^C sites in an optimal distance of about 40 to 80 base pairs [61]. The nuclease active site of the McrBC restriction endonuclease resides in its McrC subunit [62] while McrB is responsible for DNA binding and GTP hydrolysis [63, 64]. The C330A mutation in R6-DP1^R^ is not in the McrB domain responsible for recognition of methylated DNA which was shown to reside in the first 161 residues of the protein [65, 66]. The mutation is instead located in the GTPase domain located at the C-terminus of McrB, in a region conserved between several McrB sequences [67]. Interestingly, targeted mutagenesis of conserved polar amino acids to an alanine residue within the conserved region in *Escherichia coli* translated into an array of phenotypes going from complete inactivation to impaired GTP or DNA binding and even enhanced GTPase activity [67]. The equivalent residue to C330 in R6-DP1^R^ was not part of the sites targeted by the mutagenesis however and it is not possible to infer about a possible phenotype at the moment. Still, the Dp-1 genome is resistant to several type II restriction enzymes, suggesting the presence of modified bases, and it will be interesting to further study the impact of the mutated version of McrBC on Dp-1 DNA.

Finally, every lytic pneumophage studied to date (the omega phages in [27] and the SOCP and Dp-1 phages herein) are inhibited by the pneumococcal capsule and it is puzzling how these phages can thrive in natural settings among encapsulated clinical isolates. Many encapsulated pneumococcal strains also carry prophages and were thus infected by phages at some point [19–21]. On the other hand, pneumococcal strains that lack a capsule have been isolated from conjunctivitis cases [68]. The conversion between encapsulated and unencapsulated states is not uncommon in *S. pneumoniae* however and may be an important factor in population dynamics [69] and favor phage infection. Alternatively, the nasopharynx is host to the related *Streptococcus mitis* which was shown to support the replication of pneumophages, at least in the case of SOCP and Dp-1 [25]. Interestingly, non-typeable strains of *S. pneumoniae* (which include those lacking a capsule) were shown to have significantly higher probabilities to act as DNA donor in DNA recombination events compared to strains with well-defined capsule types [70]. Whether this is due to phage-mediated lysis is not known but it is worrying that non-typeable strains of *S. pneumoniae* also appear to be highly enriched in antibiotic resistance alleles [71]. It might thus be worth assessing whether sensitivity to lytic phages makes unencapsulated (non-typeable) strains a potential major reservoir to enhance the flow of resistance genes.

In conclusion, this study reported on the use of whole genome sequencing to expedite the identification of novel pneumococcal genes involved in phage-host interactions. It also suggested that different host factors are involved in the replication of phages belonging to different phage families.

## Methods

### Amplification, phage titer and adsorption of SOCP and Dp-1

Pneumophages SOCP [25] and Dp-1 [12] were obtained from the Félix d’Hérelle Reference Center for Bacterial Viruses (www.phage.ulaval.ca). Amplification and purification of bacteriophages was done on *S. pneumoniae* R6 WT as previously described for SOCP [25] and Dp-1 [23]. Phage titers were determined on *S. pneumoniae* grown in filtrated BHI+ (BHI medium supplemented with 0.25 mM CaCl_2_, 0.2 mM MgCl_2_, 8.0 μM MnCl_2_, 5 ng/mL of choline chloride and 50 mM Tris pH 7.5) at 35 °C in a 5 % CO_2_ atmosphere. When cell growth reached an OD_600_ of 0.12 a volume of 2 mL was taken and spread on agarose plates prepared by mixing equal volumes of 1 % agarose and 2× filtrated BHI+ supplemented with 50 μg ml^−1^ catalase and 0.4 % of glycine. Plates were left to stand for 5 min before the excess liquid was drained and left to dry for 10 min. Purified SOCP and Dp-1 were serially diluted in BHI and 5 μL was spotted on the bacterial top and left to dry for 10 min. The plates were incubated overnight at 35 °C under a 5 % CO_2_ atmosphere. Plaques were counted, and the phage titer was determined. EOP was calculated by dividing the phage titer (in plaque forming units (PFU) per mL) on the test strain by the phage titer in PFU per mL on the *S. pneumoniae* R6 WT indicator strain. Adsorption of bacteriophages SCOP1 and Dp-1 was determining from independent triplicates as previously described [25].

### Isolation of *S. pneumoniae* R6 bacteriophage insensitive mutants

*S. pneumoniae* R6 was grown in BHI+ supplemented with 0.4 % glycine to an OD_600_ of 0.4 under a 5 % CO_2_ atmosphere at 35 °C. A 1 mL aliquot was mixed with 100 uL of purified SOCP or Dp-1 (10^9^ PFU, multiplicity of infection of 10) and 50 μg/mL of catalase. After a 10 min incubation, the mixture was embedded in 10 mL of 0.7 % low melting point agarose in BHI+ supplemented with 0.4 % glycine. This top agarose was poured onto a 1 % BHI+ bottom agar supplemented with 0.4 % glycine and incubated overnight at 35 °C under a 5 % CO_2_ atmosphere. Seven and six colonies resistant to Dp-1 and SOCP were obtained, respectively. Resistant colonies were picked out using a sterile toothpick, spread a few times on TSA blood agar and confirmed for phage resistance as described above. All clones had similar levels of resistance to the phage used for their selection and two resistant clones (one for Dp-1 and another for SOCP) were randomly chosen for whole-genome sequencing.

### Whole genome sequencing

Genomic DNA was extracted from mutants *S. pneumoniae* R6-SOCP^R^ and R6-DP1^R^ using the Wizard Genomic DNA Purification Kit (Promega) according to the manufacturer’s instructions. Whole genome sequencing was performed using a 454 Life Sciences GS-FLX system (Roche). Genome sequencing, assemblies and comparative analyses were performed at the Institute for Integrative Systems Biology of Université Laval. Both assemblies covered >99 % of the *S. pneumoniae* R6 reference genome with a mean coverage depth of 53-fold and 47-fold for R6-DP1^R^ and R6-SOCP^R^, respectively. The detection of single nucleotide polymorphisms was performed using samtools (version 0.1.18), bcftools (distributed with samtools) and vcfutils.pl (distributed with samtools) [72]. All mutations deduced from massively parallel sequencing had at least 25-fold coverage and were confirmed by PCR amplification and conventional DNA sequencing. The sequencing reads are available on the Sequence Read Archive database under the study number PRJEB9347 and sample accession ERS719580 and ERS7195801 for R6-DP1^R^ and R6-SOCP^R^, respectively.

### RNA sequencing

Total RNA was isolated from *S. pneumoniae* R6^Δspr0058^ and R6 WT grown to mid-log phase in BHI using the Qiagen RNeasy Mini Kit (Qiagen) according to the manufacturer’s instructions. RNAs were quantified using 2100 BioAnalyzer RNA6000 Nano chips (Agilent) and 1 μg of total RNA was treated with Ribo-Zero™ rRNA Removal Kits (Epicentre). RNA-seq libraries were produced from 50 ng of rRNA-depleted samples using the ScriptSeq™ v2 RNA-Seq Library Preparation Kit (Epicentre). The libraries were analyzed using 2100 BioAnalyser High Sensitivity DNA chips and quantified by PicoGreen. The libraries were pooled, diluted to 8 pM and sequenced on an Illumina MiSeq system using a 250 bp paired-ends reads protocol. Sequence reads from each strain were filtered based on quality score using Trimmomatic [73] and aligned to the genome of *S. pneumoniae* R6 using the software bwa with default parameters [74]. A total of 3,843,210 and 3,245,555 reads derived from *S. pneumoniae* R6 WT and R6^Δspr0058^ mapped to the *S. pneumoniae* R6 reference genome, respectively. The maximum number of mismatches was 4 and the seed length was 32. Transcripts were assembled from the alignment files by using the Cufflinks pipeline [75]. Differential gene expression was computed with CuffDiff and genes with a false-discovery rate-adjusted *p*-value ≤ 0.05 were considered as differentially expressed.

### DNA transformation and gene inactivation

PCR fragments containing the mutations of interest were amplified using the Phusion High-Fidelity Polymerase (NEB) and primers listed in Additional file 5 at a final concentration of 0.5μM. DNA fragments were amplified by 35 PCR cycles each made of 10 s denaturation, 20 s annealing and 30 s (for short 500bp PCR fragments) or 3 min (for long PCR fragments of 5kb) polymerisation (with an initial denaturation of 2 min and a final extension of 10 min). PCR fragments were purified using the QIAquick PCR purification kit (QIAGEN). PCR fragments were co-transformed in *S. pneumoniae* competent cells along with 100ng of a short *rpsL* PCR fragment (500bp) amplified from *S. pneumoniae* CP1250 [40]. This co-transformed fragment is coding for a ribosomal protein S12 variant (Lys57Thr) conferring resistance to streptomycin that was used as a surrogate marker for the selection of transformants as previously described [35]. Competent cells were obtained by the dilution of an overnight *S. pneumoniae* culture 1:100 in C + Y medium, pH 6.8 (ref 47 FF). The diluted cultures were grown up to the onset of exponential phase before being concentrated ten times and frozen in C + Y, pH 6.8, 15 % glycerol. For transformation, competent cells were thawed on ice, diluted ten times with C + Y medium, pH 7.8, and complemented with 2 μg/mL of competence stimulating peptide 1 before being incubated for 15 min at 35 °C under a 5 % CO_2_ atmosphere. DNA was added to a final concentration of 2 μg/mL and the cultures were incubated for 1 h at 30 °C. Finally, the cultures were switched to 35 °C under a 5 % CO_2_ atmosphere for 1 h before being plated on CAT agar supplemented with 150μg/mL streptomycin. The plates were incubated for 48 h at 35 °C under a 5 % CO_2_ atmosphere and the resistant colonies were picked for further studies. Inactivation of spr0058 by insertional duplication mutagenesis in *S. pneumoniae* R6 was performed by cloning the middle section of spr0058 (using primers listed in Additional file 5) into the nonreplicative vector pFF6 as previously described [35]. The resulting plasmid was transformed into *S. pneumoniae* R6 as described above (without the need for *rpsL* co-transformation) and transformants were selected on CAT agar supplemented with 600μg/mL kanamycin.

### Replication of SOCP and Dp-1

*S. pneumoniae* R6 was infected with SOCP or Dp-1 at a MOI of 0.1. Total DNA (i.e. *S. pneumoniae* genomic DNA together with phage DNA) was isolated at different time points after infection using the Wizard® Genomic DNA Purification kit (Promega) following manufacturer’s instructions. Extracted DNA was digested with SspI, size-separated by electrophoresis, transferred to Nylon membrane and hybridized with [α-^32^P]dCTP-labeled probes according to standard protocols [76]. Probes were obtained by PCR amplification of the holin gene from phage SOCP or Dp-1 and from the *S. pneumoniae* gene spr1443 using primers listed in Additional file 5.

### Electron microscopy

Pneumococci were grown in BHI to an OD of 0.2. Cells were washed in 1× PBS, suspended in fixation buffer (0.1 M Cacodylate pH7.4, 2 % glutaraldehyde) and incubated at 4 °C overnight. Specimen were prepared and analyzed using standard procedure by the Plate-forme d’Imagerie Moléculaire & Microscopie of the Institute for Integrative Systems Biology of Université Laval using a Transmission Electron Microscope model JEOL 1010 at 100000× magnification. For each sample, cell wall thickness was measured from 30 bacteria (2 measures per bacteria) using ImageJ.

### Quellung reaction

Quellung reaction was performed using pneumococcal type 2 antisera from the Statens Serum Institute as described in manufacturer’s protocol. Samples were visualised under oil immersion using a Nikon Eclipse TE300 microscope and a 100× objective.

## Availability of supporting data

Sequencing reads have been deposited at the EBI SRA database under the study accession number PRJEB9347.

## Additional files

Additional file 1: Figure S1. Growth kinetics of *S. pneumoniae* R6 WT, R6-DP1^R^ and R6-SOCP^R^. The growth of *S. pneumoniae* R6 WT (black dashed line), R6-DP1^R^ (light grey line) and R6-SOCP^R^ (dark grey line) in BHI was monitored at one hour intervals for a period of 11 h. Data are expressed as the mean of three independent experiments. (TIFF 122 kb) Additional file 2: Figure S2. Cell wall thickness of *S. pneumoniae* R6 WT, R6-SOCP^R^ and R6-DP1^R^. Electron micrographs of *S. pneumoniae* R6 WT (A), R6-SOCP^R^ (B) and R6-DP1^R^ (C) at 100000× magnification. (D) Mean cell wall thickness for *S. pneumoniae* R6 WT (black), R6-SOCP^R^ (light grey) and R6-DP1^R^ (dark grey) measured from 30 bacteria with two measurements per bacteria. (TIFF 4146 kb) Additional file 3: Figure S3. Gene expression profiling in *S. pneumoniae* R6^Δspr0058^. Gene expression was compared between *S. pneumoniae* R6 WT and R6^Δspr0058^ by RNA-seq. The dots represent the 2043 genes from the *S. pneumoniae* R6 genome and their level of expression in R6 WT and R6^Δspr0058^ (represented in terms of fragments per kilobase of transcript per million fragments mapped) is indicated on the x- and y-axis, respectively. (TIFF 151 kb) Additional file 4.
**Genes with an altered expression upon inactivation of spr0058 as determined by RNAseq.** (PDF 128 kb) Additional file 5.
**PCR primers used in this study.** (PDF 23 kb)

## Abbreviations

EOP: Efficiency Of Plaquing
MOI: Multiplicity Of Infection
PFU: Plaque Forming Unit
RNA-seq: RNA sequencing
rRNA: Ribosomal RNA
WT: Wild-type
