# Supplementary figures and images for: A genomic approach to understand interactions between Streptococcus pneumoniae and its bacteriophages
Source: BMC Genomics. 2015 Nov 18;16:972. doi: 10.1186/s12864-015-2134-8 (PMC4652380; doi:10.1186/s12864-015-2134-8)

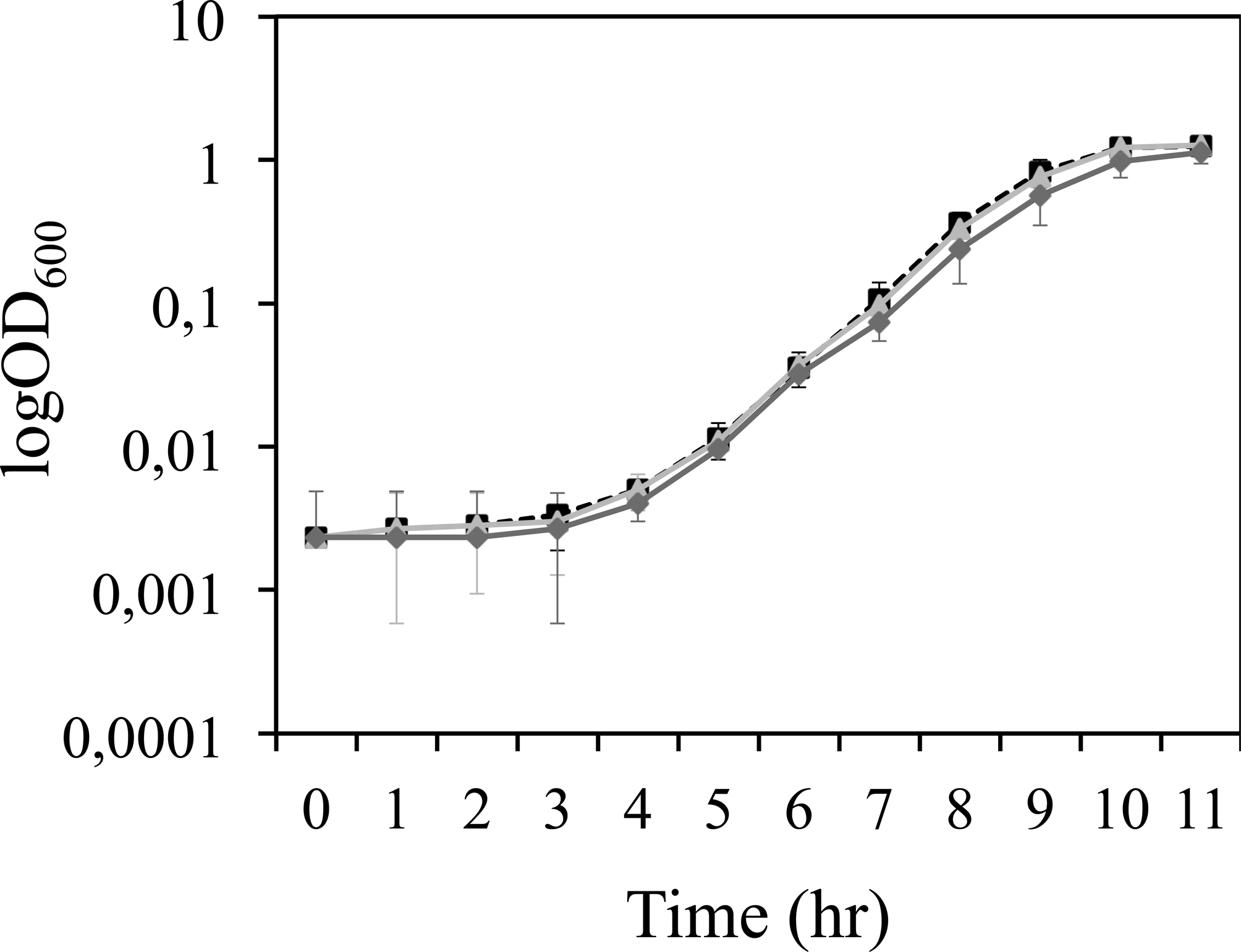

Supplement: Additional file 1: Figure S1. — Growth kinetics of S. pneumoniae R6 WT, R6-DP1R and R6-SOCPR. The growth of S. pneumoniae R6 WT (black dashed line), R6-DP1R (light grey line) and R6-SOCPR (dark grey line) in BHI was monitored at one hour intervals for a period of 11 h. Data are expressed as the mean of three independent experiments. (TIFF 122 kb) [file 12864_2015_2134_MOESM1_ESM.tif]

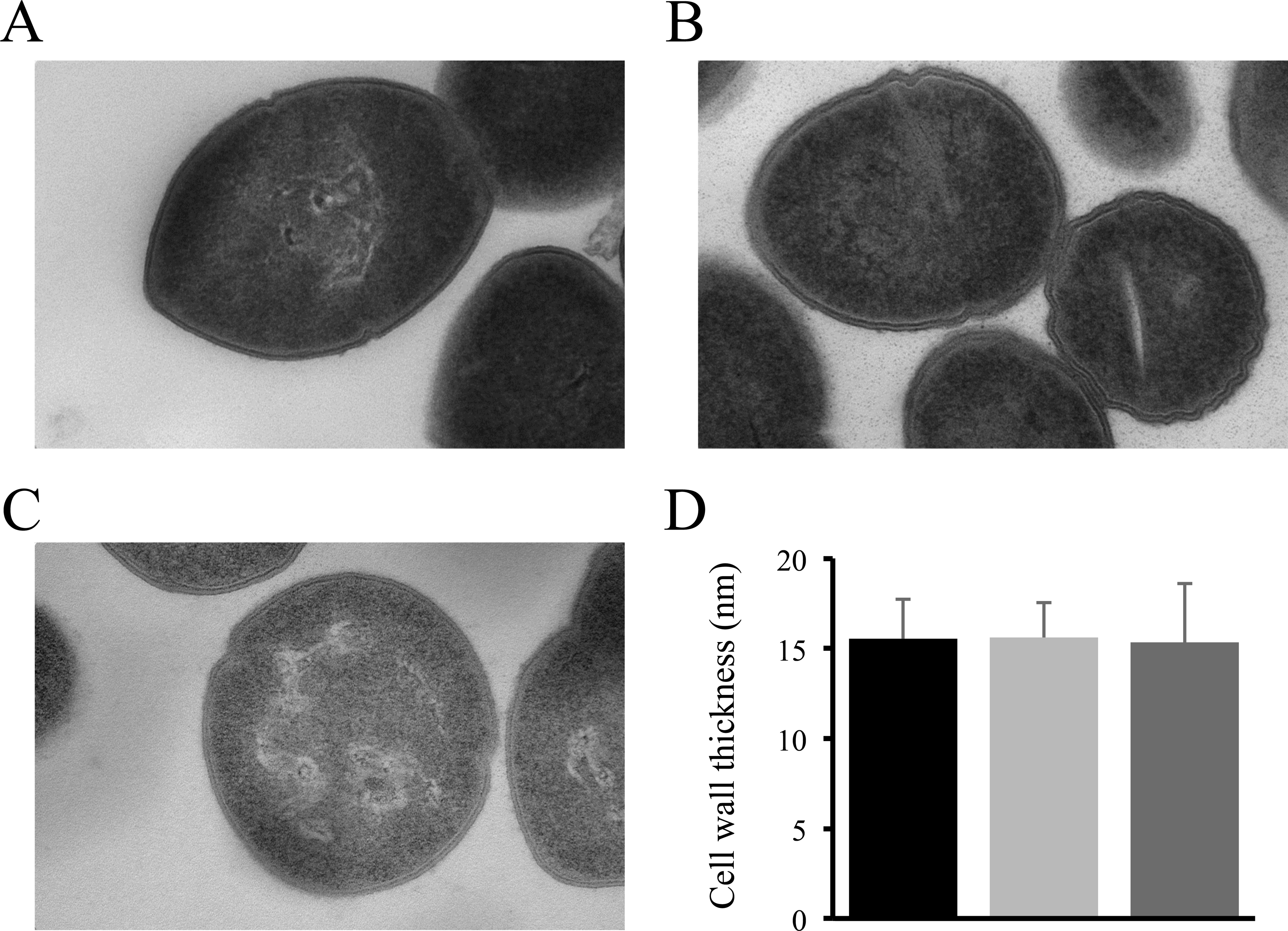

Supplement: Additional file 2: Figure S2. — Cell wall thickness of S. pneumoniae R6 WT, R6-SOCPR and R6-DP1R. Electron micrographs of S. pneumoniae R6 WT (A), R6-SOCPR (B) and R6-DP1R (C) at 100000× magnification. (D) Mean cell wall thickness for S. pneumoniae R6 WT (black), R6-SOCPR (light grey) and R6-DP1R (dark grey) measured from 30 bacteria with two measurements per bacteria. (TIFF 4146 kb) [file 12864_2015_2134_MOESM2_ESM.tif]

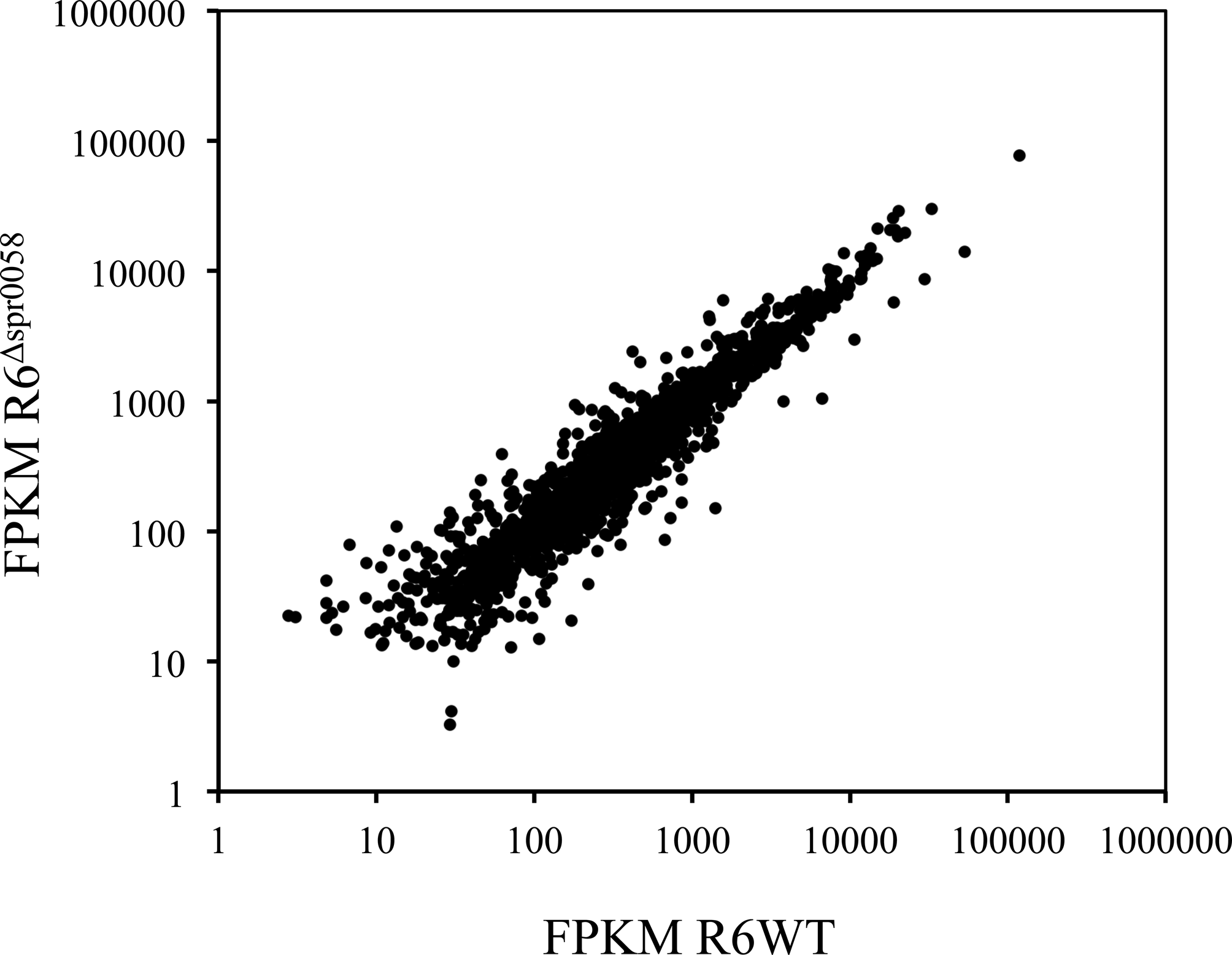

Supplement: Additional file 3: Figure S3. — Gene expression profiling in S. pneumoniae R6Δspr0058. Gene expression was compared between S. pneumoniae R6 WT and R6Δspr0058 by RNA-seq. The dots represent the 2043 genes from the S. pneumoniae R6 genome and their level of expression in R6 WT and R6Δspr0058 (represented in terms of fragments per kilobase of transcript per million fragments mapped) is indicated on the x- and y-axis, respectively. (TIFF 151 kb) [file 12864_2015_2134_MOESM3_ESM.tif]
