# Supplementary material for: A genomic approach to understand interactions between Streptococcus pneumoniae and its bacteriophages
Source: BMC Genomics. 2015 Nov 18;16:972. doi: 10.1186/s12864-015-2134-8 (PMC4652380; doi:10.1186/s12864-015-2134-8)
Supplement: Additional file 4. — Genes with an altered expression upon inactivation of spr0058 as determined by RNAseq. (PDF 128 kb) [file 12864_2015_2134_MOESM4_ESM.pdf]

Additional file 4

Table S1. Genes with an altered expression upon inactivation of spr0058 as determined by RNAseq.

R6_geneID	Function	FPKM[a] R6WT	FPKM[a] $R6^{\Delta spr0058}$	log2 (KO/WT)	*p* value	*q* value
		Genes downregulated in $R6^{\Delta spr0058}$				
**spr0081**	ABC transporter permease	377.482	84.0558	-2.16699	0.000181602	0.00931757
**spr0082**	ABC transporter permease	341.228	57.1887	-2.57693	1.47E-05	0.00113125
**spr0083**	ABC transporter substrate-binding protein	259.203	30.1397	-3.10434	2.14E-07	3.05E-05
**spr0127**	hypothetical protein	5866.42	1367.38	-2.10107	0.000320174	0.015254
**spr0128**	hypothetical protein	2841.73	676.937	-2.06967	0.000470955	0.0204865
**spr0440**	endo-beta-N-acetylglucosaminidase	110.743	25.8047	-2.10151	0.000146839	0.0081618
**spr1724**	single-stranded DNA-binding protein	916.763	172.564	-2.40941	5.05E-05	0.00325931
**spr1837**	bifunctional acetaldehyde-CoA/alcohol dehydrogenase	8237.01	1512.64	-2.44506	1.15E-07	1.91E-05
**spr1856**	hypothetical protein	139.4	19.6117	-2.82944	0.000188588	0.00943411
**spr1858**	hypothetical protein	571.991	83.9454	-2.76847	5.44E-06	0.000494609
**spr1859**	hypothetical protein	154.251	32.4824	-2.24755	0.00085038	0.0340322
**spr1861**	competence protein CglD	313.504	51.8821	-2.59518	3.56E-05	0.00245447
**spr1862**	competence protein CglC	714.751	134.909	-2.40545	9.97E-05	0.00586808
**spr1863**	competence protein CglB	380.283	56.4995	-2.75076	3.34E-06	0.000318369
**spr1864**	competence protein CglA	583.43	88.358	-2.72313	2.69E-06	0.000283159
**spr1936**	transketolase, C-terminal subunit	1426.9	273.37	-2.38396	9.15E-06	0.000796012
**spr1937**	transketolase n-terminal section	1232.83	238.558	-2.36955	1.08E-05	0.000904189
**spr1938**	PTS system ascorbate-specific transporter subunit IIC	808.009	164.622	-2.29522	1.92E-05	0.00140344
**spr1939**	PTS system IIB component	1402.61	285.359	-2.29726	0.000107708	0.00615784
**spr1940**	hypothetical protein	959.604	272.056	-1.81853	0.000947187	0.0371632
**spr1949**	glycosyl hydrolase-related protein	103.522	10.9756	-3.23756	1.27E-07	1.95E-05
**spr1950**	ROK family protein	99.2467	13.0581	-2.92608	1.28E-05	0.00102654
**spr1951**	hypothetical protein	98.0277	11.6118	-3.0776	3.28E-07	4.37E-05
**spr1952**	hypothetical protein	71.9572	12.4131	-2.53527	9.17E-05	0.00556239
**spr1966**	hypothetical protein	67.7091	13.9362	-2.28051	0.000177384	0.00931757
**spr1967**	PTS system IID component	193.493	26.2447	-2.88218	3.34E-06	0.000318369
**spr1968**	PTS system IIC component	180.5	28.3971	-2.66818	1.96E-05	0.00140344
**spr1969**	PTS system IIB component	649.476	151.453	-2.1004	0.000375416	0.01747
**spr1970**	PTS system IIA component	327.962	74.8861	-2.13076	0.000623509	0.0259925
**spr1987**	hypothetical protein	3327.55	314.128	-3.40504	5.13E-08	9.33E-06
**spr1988**	glycerol uptake facilitator protein	825.036	110.367	-2.90215	7.18E-07	8.45E-05
**spr1989**	glycerol-3-phosphate dehydrogenase	1521.66	236.257	-2.68722	2.07E-06	0.000230101

**spr2013**	helicase	17.531	2.65317	-2.72412	0.000734292	0.0299861
Genes upregulated in R6$^{\Delta spr0058}$						
**spr0059**	Beta-galactosidase	263.543	4437.5	4.07364	6.98E-09	1.55E-06
**spr0060**	PTS system IIB component	104.66	1676.63	4.00179	2.37E-11	9.47E-09
**spr0061**	PTS system IIC component	235.929	2672.44	3.50174	2.57E-10	8.56E-08
**spr0062**	PTS system IID component	150.757	1645.83	3.44852	5.11E-10	1.28E-07
**spr0063**	PTS system IIA component	179.587	2353.97	3.71234	3.17E-10	9.08E-08
**spr0064**	tagatose-6-phosphate isomerase	102.829	1489.46	3.85647	5.29E-12	3.53E-09
**spr0065**	aldose 1-epimerase	57.9243	882.493	3.92934	2.16E-11	9.47E-09
**spr0092**	NAD-dependent epimerase/dehydratase family protein	340.426	1644.1	2.27189	7.16E-05	0.00447493
**spr0310**	glucan 1,6-alpha-glucosidase	177.718	824.112	2.21325	3.95E-05	0.00263421
**spr0328**	cell wall surface anchor family protein	32.4643	890.032	4.77693	1.40E-14	2.80E-11
**spr0528**	VncR, response regulator	167.727	646.49	1.94651	0.000599926	0.0255415
**spr0529**	VncS, histidine kinase	162.836	662.614	2.02475	0.00016685	0.00902344
**spr0668**	PTS system IIABC component	83.8985	316.363	1.91486	0.000410764	0.0186804
**spr1324**	thiamine biosynthesis protein	133.511	524.898	1.97508	0.000433737	0.0192868
**spr1698**	dextran glucosidase	45.9816	750.799	4.0293	2.47E-12	2.47E-09
**spr1699**	trehalose-specific PTS system IIBC component	33.3452	316.49	3.24661	2.22E-08	4.45E-06
**spr1914**	hypothetical protein	64.4146	445.444	2.78978	0.000283421	0.0138323
**spr1915**	hypothetical protein	272.417	1824.6	2.74369	5.77E-07	7.21E-05

[a]Fragments Per Kilobase of transcript per Million fragments mapped.
