# Supplementary material for: A genomic approach to understand interactions between Streptococcus pneumoniae and its bacteriophages
Source: BMC Genomics. 2015 Nov 18;16:972. doi: 10.1186/s12864-015-2134-8 (PMC4652380; doi:10.1186/s12864-015-2134-8)
Supplement: Additional file 5. — PCR primers used in this study. (PDF 23 kb) [file 12864_2015_2134_MOESM5_ESM.pdf]

Additional file 5

Table S2. PCR primers used in this study.

**PrimerID[a]**	**Target gene**	**Sequence (FW / RV)**
spr0058_500bp	spr0058	TCATCTCTGGGCAGTTTAGC / GCCAGTTGATTCGAGTTTGG
spr0093_500bp	spr0093	ATGACCTCTATCACAGCG / CTCTAAGGGTTTGTGAGC
spr0290-0291_500bp	intergenic	CCGGTGATGAGAGCAATTTT/ CTGCTGACATTCCTTCCACA
spr0897_500bp	spr0897	AATTGAGTATATCCGCAAGG / TCTGAGTGATATCATCTACC
spr1130_500bp	spr1130	CTTAGAAATCTCCCCACGATTG/ CCAGTATCAAATGGGGATGG
spr1191_500bp	spr1191	GATATCTTCTCGAGAACCAG / CTCCATGATATTACAGATGG
spr1443_500bp	spr1443	TTCGCAACTCATCAAACAGG / TTAGTTCATCTCCTTTCTAAC
spr1445_500bp	spr1445	ACTCTTGAAGCCCAACAAGC/ GGAGAGCACCCTCGTTATCA
spr1453-1454_500bp	box element	GTCAAACATGGCTGGTAGGG/ TGTGAACAAGGGTTGGAATG
spr1584_500bp	spr1584	CACGAAGCTCGCGAATCACG / GGACAAGCTGAGCGTGAAGC
spr1777_500bp	spr1777	GTCATGTAGGCAACGATT / CTCGTATCCACGATGCTAGC
spr1923_500bp	spr1923	GGAGCTTGGATCCACTTGTG / AGATTTCGGCCTCTCTGGTC
spr0058_5kb	spr0058	ATGATACGGTTGGCACCTTCC / GGTACCTCGTTAAGTGCTTGC
spr0093_5kb	spr0093	TGTTGCAACAGGCGCTACAGG / GCCTTCTTGCCACTCTTCTCC
spr0290-0291_5kb	intergenic	AGTTGGTAAAGGGCACCAGA/ TCGGAGTAGAGAGGGCAAGA
spr0897_5kb	spr0897	GACGAGCAGATGCGTATCAA / ACAAGGTGTCCAACCAAAGC
spr1130_5kb	spr1130	CCACCTTTAGCCAGTTGCTC/ GGCCTGGTTAACTGGAATCA
spr1191_5kb	spr1191	GCTGGCGATAAGAGTTCGAC / TGTGAGCAAGTGCAAAGACC

spr1443_5kb	spr1443	ACTTCCCATGACTTTCGGACC / CATCCATACCGATACCATGCC
spr1445_5kb	spr1445	AATCGCTCTCGCTTTGTCAT/ GCTGGTGCAGGTTGTACCTT
spr1453-1454_5kb	box element	TCCACATCACGCAAGCTATC/ AGTTTGGCAAAGTCCACAGG
spr1584_5kb	spr1584	CCTGATTCCCTTTCCTATCCC / GACTGCCTGATATTCCAGACC
spr1777_5kb	spr1777	TTTGCAGAGCCGTGCCTTACC / GCACGAGGGCTCATATCAAGG
spr1923_5kb	spr1923	AGTCAGTAACCATACCTACGG / ATCAGCTTGTCTATGGTACCC
KO_spr0058	spr0058	ATATTGTCCGCCAACAAGGA / ACTGAAGCCGCATGTTCT
KO_cps2C	SPD_0317	GGCACTTGTGACAGGTGCGG / CTTCAGGACGTTTCACACGG
Southern_Holin_Cp-1	holin	GCTAGAAGTTGCTAAAGG/ GCAATTCTTCCTGTACTC
Southern_Holin_Dp-1	holin	ATGAAACTATCTAACGAAC/ ATTCCACCTCATTGTTTTG

[a]Primers 500bp were used for the confirmation of mutations. Primers 5kb were used for resistance reconstruction.
